# Depolarization-induced depression of inhibitory transmission in cerebellar Purkinje cells

**DOI:** 10.1002/phy2.61

**Published:** 2013-08-22

**Authors:** Hiromasa Satoh, Lihui Qu, Hidenori Suzuki, Fumihito Saitow

**Affiliations:** 1 Department of Pharmacology, Nippon Medical SchoolTokyo, 113-8602, Japan; 2 Department of Physiology, Harbin Medical University-DaqingHeilongjiang, 163319, China; 3 Japan Science and Technology Agency, CRESTTokyo, 102-0075, Japan

**Keywords:** Ca^2+^/Calmodulin-dependent protein kinase II, cerebellum, chloride ion, GABAergic synaptic transmission, synaptic plasticity

## Abstract

Several forms of depolarization-induced plasticity in inhibitory transmission have been reported to occur in cerebellar Purkinje cells (PCs), namely depolarization-induced suppression of inhibition (DSI), depolarization-induced potentiation of inhibition (DPI), and rebound potentiation (RP). Here, we describe another form of synaptic plasticity for gamma-amino butyric acid (GABA)ergic transmission in PCs. Immediately following depolarization trains in a PC, evoked inhibitory postsynaptic currents (eIPSCs) changed their direction from outward to inward currents under a recording condition in which eIPSCs were elicited as an outward current. Subsequently, the eIPSC amplitude remained depressed (depolarization-induced depression of inhibition [DDI]) for more than 20 min under the blockade of cannabinoid and N-methyl-D-aspartic acid (NMDA) receptor-mediated DSI and DPI, respectively. This DDI was completely abolished by intracellular infusion of the fast Ca^2+^-chelating agent BAPTA and by inhibition of Ca^2+^/calmodulin-dependent protein kinase II (CaMKII). Furthermore, DDI was strongly suppressed by calcium-activated chloride channel (CaCC) blockers, while an inhibitor of cation-chloride cotransporters (CCCs) partially blocked DDI during the early phase. Exogenous GABA-induced inhibition of spontaneous spike activity was attenuated in ∼50% of the PCs by climbing fiber stimulation-induced depolarization. These results suggest that activation of both CaCCs and CCCs was necessary for alteration of [Cl^-^]_i_ after activation of CaMKII following elevation of [Ca^2+^]_i_ in PCs. DDI may provide another mechanism for regulation of inhibitory inputs to PCs within the neuronal networks of the cerebellar cortex.

## Introduction

Gamma-amino butyric acid (GABA) is the major inhibitory neurotransmitter in the central nervous system, with actions mediated by its cognate receptors, GABA_A_, GABA_B_, and GABA_C_. GABA_A_ receptors comprise a ligand-gated ion channel through which chloride ions (Cl^−^) pass (Bormann 1988, 2000). It is generally accepted that neuronal intracellular Cl^−^ concentration ([Cl^−^]_i_) is primarily regulated by cation-chloride cotransporters (CCCs), which function electroneutrally, by Na^+^-K^+^−2Cl^−^ cotransporters (NKCCs), which normally accumulate Cl^−^ (Payne et al. 2003; Yamada et al. 2004), and by K^+^−Cl^−^ cotransporters (KCC2s), which normally extrude Cl^−^ (Lu et al. 1999; Rivera et al. 1999, 2005). A similar phenomenon has been reported in Purkinje cells (PCs), whereby the intracellular concentration of calcium ions ([Ca^2+^]_i_) is transiently elevated by GABA_A_ receptor activation in newborn rats (Eilers et al. 2001). As with the developmental regulation of [Cl^−^]_i_, activity-induced changes in [Cl^−^]_i_ have been observed following tetanic stimulation (Kaila et al. 1997), epileptic activity (Rivera et al. 2004), rebound burst activity (Wang et al. 2006), and repeated postsynaptic spiking (Fiumelli et al. 2005). In particular, it has been proposed that modulation of KCC2 expression or function is crucial for both neuronal Cl^−^ homeostasis and GABAergic transmission in neuronal networks. Furthermore, calcium-activated chloride channels (CaCCs) are known to play important roles in cellular physiology, such as transduction in sensory neurons (Hartzell et al. 2005). In olfactory receptor neurons, Cl^−^ efflux through CaCCs serves as an amplification mechanism for odorant-activated currents (Lowe and Gold 1993; Kleene 1997).

Synaptic plasticity, a cellular substrate of learning and memory, relies on the precise regulation of neuronal excitability. Depolarization of PCs increases [Ca^2+^]_i_, which triggers various plastic responses of inhibitory transmission. These changes can be classified as rebound potentiation (RP; [Kano et al. 1992; Hashimoto et al. 1996;]), depolarization-induced suppression of inhibition (DSI; [Kreitzer and Regehr 2001;]), and depolarization-induced potentiation of inhibition (DPI; [Duguid and Smart 2004]). DSI and DPI involve retrograde actions of endocannabinoids and glutamate, respectively, which are released from PCs. These retrograde messengers act on their presynaptic receptors to regulate presynaptic transmitter release. In contrast, RP is induced by postsynaptic molecular mechanisms. The activation of Ca^2+^/calmodulin-dependent protein kinase II (CaMKII) is necessary for RP induction after [Ca^2+^]_i_ increase (Kano et al. 1996). The activity of CaMKII is regulated by protein phosphatase-1 (PP-1) via the cAMP-dependent protein kinase (PKA)-mediated signal pathway, which is activated by G_i/o_-coupled GABA_B_ receptors (Kawaguchi and Hirano 2000, 2002). Furthermore, activation of glutamate receptor mGluR1 counteracts GABA_B_ receptor activity and contributes to RP induction by PKA activation, PP-1 downregulation, and CaMKII upregulation (Sugiyama et al. 2008). The majority of the above mentioned findings were obtained in electrophysiological studies conducted using high-Cl^−^ pipette conditions and exogenously applied GABA.

In this study, we observed the depolarization-induced plasticity of inhibitory synapses in PCs under more physiological conditions; we recorded stimulation-evoked inhibitory postsynaptic currents (eIPSCs) using a low-Cl^−^ pipette solution. Here, we report the PC depolarization-induced depression of eIPSC amplitude. This phenomenon depends on the postsynaptic [Cl^−^]_i_, which is increased via the activation of CaCCs and, in part, the activation of NKCCs. These changes in [Cl^−^]_i_ required CaMKII activation following [Ca^2+^]_i_ elevation via voltage-dependent Ca^2+^ channels (VDCCs), activated by PC membrane depolarization. Furthermore, stimulation of climbing fibers (CFs) reduced GABA-mediated inhibition of spontaneous spike activity in half of the PCs examined. Together, our findings describe a postsynaptic mechanism that is different from the well-established depolarization-induced plasticity of GABAergic inhibitory transmission in cerebellar PCs.

## Material and Methods

### Preparations

All animal experiments were approved by the Ethics Review Committee of Nippon Medical School. Parasagittal cerebellar slices (300 μm) were obtained from Wistar rats (postnatal day 11–15, either sex) as has been described previously (Saitow et al. 2005). Briefly, animals were deeply anesthetized by halothane inhalation (∼2% in air, v/v), and their brains were rapidly removed. Sections were cut using a vibratome (VT1200S; Leica, Nussloch, Germany) in an ice-cooled cutting solution containing the following (in mmol/L): 299.2 sucrose, 3.4 KCl, 0.3 CaCl_2_, 3 MgCl_2_, 10 HEPES, 0.6 NaH_2_PO_4_, and 10 glucose and bubbled with 100% O_2_ (pH adjusted to 7.4 using NaOH). The slices were maintained in an interface chamber for at least 1 h in an artificial cerebrospinal fluid (ACSF) containing the following (in mmol/L): 138.6 NaCl, 3.4 KCl, 2.5 CaCl_2_, 1.0 MgCl_2_, 21.0 NaHCO_3_, 0.6 NaH_2_PO_4_, and 10 glucose. The pH was maintained at 7.4 by bubbling with 95% O_2_ and 5% CO_2_. To assess the contribution of bicarbonate ions to GABA_A_ receptor-mediated currents, NaHCO_3_ was replaced with HEPES (HEPES-ACSF). HEPES-ACSF was bubbled with 100% O_2_, and the pH was adjusted to 7.4 with NaOH. To confirm the effect of niflumic acid (NFA) on the functions of calcium channels, we used a solution (TEA-Ba) containing the following (in mmol/L): 150 tetraethylammonium chloride, 5 BaCl_2_, 1 MgCl_2_, 10 HEPES, and 10 glucose. The TEA-Ba solution was bubbled with 100% O_2_, and the pH was adjusted to 7.4 with NaOH.

### Patch clamp recordings

Individual slices were transferred to a recording chamber mounted on the stage of an upright microscope equipped with Nomarski optics (BX61WI; Olympus, Tokyo, Japan) and an infrared CCD camera control system (C2741; Hamamatsu Photonics, Hamamatsu, Japan). The preparations were continuously superfused with oxygenated ACSF at a rate of 1.5 mL/min. All recording data were obtained at room temperature (25–27°C) except as otherwise noted. When the experiments were carried out under the condition of recording temperature at 30°C, temperature controller (TC-324B; Warner Instruments, Hamden, CT) and inline solution heater (64-0102; Warner Instruments) were used to warm the perfusate.

Unless otherwise specified, ACSF contained 10 μmol/L 6-cyano-7-nitroquinoxaline-2,3-dione (CNQX), 40 μmol/L D-(-)-2-amino-5-phosphonopentanoic acid (APV), 2 μmol/L CGP 55845, and 2 μmol/L AM 251 to block glutamatergic inputs, and GABA_B_ receptor- and CB_1_ receptor-mediated responses, respectively. Several experiments were performed in the presence of tetrodotoxin (TTX; 0.5 μmol/L) in order to block action potential-dependent synaptic transmission. For conventional whole-cell voltage clamp mode, patch pipettes with a resistance of 2.0–3.5 MΩ were filled with a low-Cl^−^ internal solution (CsMS) containing the following components (in mmol/L): 150.0 Cs-methanesulfonate, 5.0 KCl, 0.1 Cs-EGTA, 10 Na-HEPES, 3.0 Mg-ATP and 0.4 Na-GTP (pH 7.4 with CsOH, 300 mOsm). To examine the effects of intracellular infusion of a fast Ca^2+^ chelator, 1,2-Bis(2-Aminophenoxy)ethane-*N,N,N′,N′*-tetraacetic acid (BAPTA), we employed an internal solution containing the following components (in mmol/L): 150.0 Cs-methanesulfonate, 5.0 KCl, 5.0 BAPTA, 0.05 Cs-EGTA, 10 Na-HEPES, 3.0 Mg-ATP, and 0.4 Na-GTP (pH 7.4 with CsOH, 300 mOsm). In order to explore the effect of Cl^−^ concentration on depolarization-induced depression of inhibition (DDI), we used a moderate Cl^−^-containing internal solution (CsMS-mid), such that the Cl^−^ equilibrium potential was approximately −40 mV. This solution contained (in mmol/L): 125.0 Cs-methanesulfonate, 25.0 CsCl, 5.0 KCl, 0.1 Cs-EGTA, 10 Na-HEPES, 3.0 Mg-ATP, and 0.4 Na-GTP (pH 7.4 with CsOH, 300 mOsm). To measure the resting membrane potential of PCs, we used an intracellular solution (KMS) containing the following (in mmol/L): 150.0 K-methanesulfonate, 5.0 KCl, 0.1 K-EGTA, 10 Na-HEPES, 3.0 Mg-ATP, and 0.4 Na-GTP (pH 7.4 with KOH, 300 mOsm). For gramicidin-perforated patch recording, patch pipettes with a resistance of 3.5–5.0 MΩ were filled with a high-Cl^−^ internal solution (KCl) containing the following (in mmol/L): 150 KCl and 10 Na-HEPES (pH 7.35 with KOH) (Eilers et al. 2001). The ends of the pipettes were dipped in a normal (gramicidin-free) pipette solution, and then left for 3–5 min to fill the pipette tip. The pipette was then back-filled with the same internal solution, to which 50 μg/mL gramicidin had been added. A 50 mg/mL stock solution of gramicidin in DMSO was prepared freshly (no more than 2 h before recording) and sonicated for 30 sec. This stock solution was then diluted with normal pipette solution and sonicated again for 30 sec.

Membrane currents were recorded using a patch clamp amplifier (EPC-8; HEKA Electronik, Lambrecht, Germany), filtered at 3 kHz, and sampled at 5 kHz via an AD/DA converter (Digidata1322A; Molecular Devices, Sunnyvale, CA), which was controlled by the Clampex software (version 10.2; Molecular Devices). The membrane potential of PCs was held at −70.5 mV to record eIPSCs. To monitor the paired-pulse ratio (PPR), which was defined by the ratio of the second to the first IPSC amplitude, eIPSCs were evoked with an interstimulus interval of 50 msec every 15 sec by electrical stimulation (20–80 μA, 100–150 μsec) using glass electrodes (tip diameter of 1–2 μm) filled with ACSF. The series resistance, which was monitored throughout the experiment, was 5–15 MΩ and was not compensated. Data were discarded if this value changed by more than 20% during recording. The stimulation electrodes were placed on the molecular layer in order to stimulate GABAergic interneurons. The evoked IPSCs were abolished by bath application of bicuculline (10 μmol/L), indicating that these were mediated by GABA_A_ receptors.

**Figure 1:**
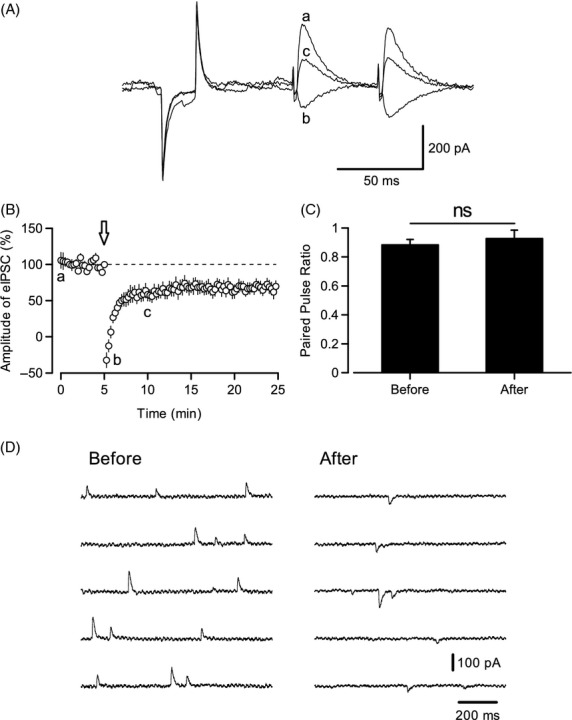
Depolarization of PCs-induced DDI. (A) Representative traces of eIPSCs recorded using a low-Cl^−^ pipette solution. A test pulse (−5 mV) was applied to monitor series resistance. a. Control response recorded as an outward current at *t* = 0; b. the response recorded immediately following the depolarizing pulses (at *t* = 5); c. the response recorded at *t* = 10 with the depression of the eIPSC amplitude. a, b, and c are represented in the timing of B. (B) Time course of the eIPSC amplitude was recorded using conventional whole-cell patch clamp (*n* = 8). Five depolarizing train pulses (−70.5 mV to −20.5 mV for 1 sec at 0.5 Hz) were applied at *t* = 5, arrow. PC depolarization caused the depression of eIPSC amplitude for more than 20 min (*n* = 8). (C) Comparison of PPR between before (*t* = 3) and after (*t* = 20) depolarization. The PPR was not significantly different (*P* = 0.5, *n* = 8). (D) Depolarization induced by CF stimulation (1 Hz × 20 times) changed the polarity of spontaneous IPSCs. Raw traces of spontaneous IPSCs immediately before and after CF stimulation. This experiment was performed in the presence of 2 μmol/L AM 251 in order to eliminate DSI.

In order to examine the effects of CF-induced depolarization on spontaneous spike generation in PCs, cell-attached recordings were obtained. For spike recording, glass electrodes with a resistance of 5.0–7.0 MΩ were filled with ACSF. During the recording of both spontaneous IPSCs (sIPSCs; Fig. 1D) and spontaneous spikes in PCs (Fig. 7), a second glass electrode was placed on the granule cell layer in order to stimulate CFs (1 Hz, 20 times). The CF response was identified as either paired-pulse depression or a set of small spikelets following a large spike (Eccles et al. 1966), and as an all-or-none response that was dependent on the stimulation intensity. The liquid junction potential was compensated for in our data set (10.5 mV for CsMS, 7.7 mV for CsMS-mid, 9.9 mV for KMS, and 4.3 mV for KCl). All data were harvested from lobule III–VIII, and were analyzed using the Clampfit (version 10.2; Molecular Devices), Kyplot (version 5.0; Kyence, Tokyo, Japan), and Igor Pro (version 5.0; WaveMetrics, Lake Oswego, OR) software. Data are summarized as means ± standard error of the mean (SEM). Unless otherwise stated, Student's *t*-test was used to assess statistically significant differences. A *P*-value of less than 0.05 was considered to indicate statistical significance.

### Drug application and chemicals

The majority of the drugs were delivered by bath application. Exogenous GABA was dissolved in ACSF and applied to the primary dendritic shaft of the recording neuron by means of a puffer pipette (3.5 psi), which was regulated by a pressure control system (PV830; World Precision Instruments, Sarasota, FL). 4,4′-Diisothiocyanatostilbene-2,2′-disulfonic acid disodium salt (DIDS) and NFA were dissolved in the pipette solution, and applied intracellularly via the recording pipette. To assess the contribution of CCCs, slices were preincubated with the CCC inhibitors bumetanide and (R-[+]-[(2-*n*-butyl-6,7-dichloro-2cyclopentyl-2,3-dihydro-1-oxo-1H-inden-5-yl)oxy]acetic acid (DIOA) for at least 1 h. The chemicals used in this study were purchased from the following sources: gramicidin D, BAPTA, CNQX, APV, KN62, DIDS, NFA, bumetanide, and DIOA from Sigma (St. Louis, MO); CGP55845 and AM 251 from Tocris Cookson (Bristol, U.K.); and TTX from Sankyo (Tokyo, Japan).

## Results

### PC depolarization induces depression of eIPSCs

The amplitude of eIPSCs was recorded as an outward current using a low Cl^−^ pipette solution, with the membrane potential maintained at −70.5 mV (Fig. 1A a). The ACSF contained CNQX (10 μmol/L), APV (40 μmol/L), CGP55845 (2 μmol/L), and AM 251 (2 μmol/L) to prevent the activation of alpha-amino-3-hydroxy-5-methyl-4-isoxazolepropionic acid (AMPA), N-methyl-D-aspartic acid (NMDA), GABA_B_, and CB_1_ receptors, respectively. Immediately following a depolarization train (5 depolarization pulses, −70.5 mV to −20.5 mV for 1 sec at 0.5 Hz), the amplitude of eIPSCs was markedly attenuated (−32.30 ± 10.18% at *t* = 5, *n* = 8, Fig. 1A and B). In most of the cells, the polarity of eIPSCs changed from outward to inward immediately following the depolarizing pulses. The amplitude of eIPSCs remained depressed for more than 20 min (62.51 ± 9.26% at *t* = 20, *n* = 8, Fig. 1A and B). In the presence of AM 251, a CB_1_ receptor antagonist, DSI did not occur, indicating that the alteration of synaptic transmission cannot be explained by retrograde messenger-mediated suppression (Kreitzer and Regehr 2001). Furthermore, DPI (which is also mediated by retrograde messengers) was abolished by the NMDA receptor antagonist APV under the same experimental conditions. It has been reported that RP is postsynaptically suppressed by GABA_B_ receptor-mediated signaling (Kawaguchi and Hirano 2000). In this case, the depression of eIPSC amplitude could not be attributed to GABA_B_ receptor activation since the ACSF contained the GABA_B_ receptor antagonist CGP55845 throughout the experiments, with the exception of the experiments shown in Figures 1D and 7.

Next, to determine whether this depolarization-induced depression of eIPSCs was mediated by a presynaptic or postsynaptic mechanism, we compared the amplitude and PPR of eIPSCs (evoked at 50-msec intervals) before (at *t* = 3) and after (at *t* = 20) depolarization (Fig. 1B). This depression revealed a significant reduction in eIPSC amplitudes (90.19 ± 4.37% before, and 62.51 ± 9.26% after depolarization, *n* = 8, *P* < 0.05), but this was not associated with any alterations in the PPR (0.88 ± 0.04 before and 0.93 ± 0.06 after depolarization, *n* = 8, *P* = 0.47, Fig. 1C). We observed these eIPSC depressions in 66.7% of the recorded PCs (8 of 12 cells). Next, we examined whether the more physiological depolarization evoked by CFs also influenced GABAergic sIPSCs. As shown in Figure 1D, the polarity of sIPSCs was altered after CF stimulation (1 Hz, 20 times) under current-clamp condition, in a similar manner to that shown in Figure 1A b. This experiment was performed in the presence of AM 251 (2 μmol/L) to eliminate any DSI. Data were recorded for 30 sec before and after the depolarization of PCs. This change in the polarity of sIPSCs was consistent with the early phase of depression of eIPSC amplitude induced by depolarizing voltage-steps, as shown in Figure 1A and B. This indicates that depolarization by CF stimulation also elicited, at least partially, the depolarization-induced depression of GABAergic transmission onto PCs. CF-induced depression of sIPSC amplitude were observed in 66.7% of the recorded PCs (6 out of 9 cells). Taken together, our results suggest that depression of eIPSC amplitude is mediated by a postsynaptic mechanism distinct from DSI and GABA_B_ receptor-mediated modulation. On the basis of our findings, we refer to this phenomenon as DDI hereafter.

**Figure 2:**
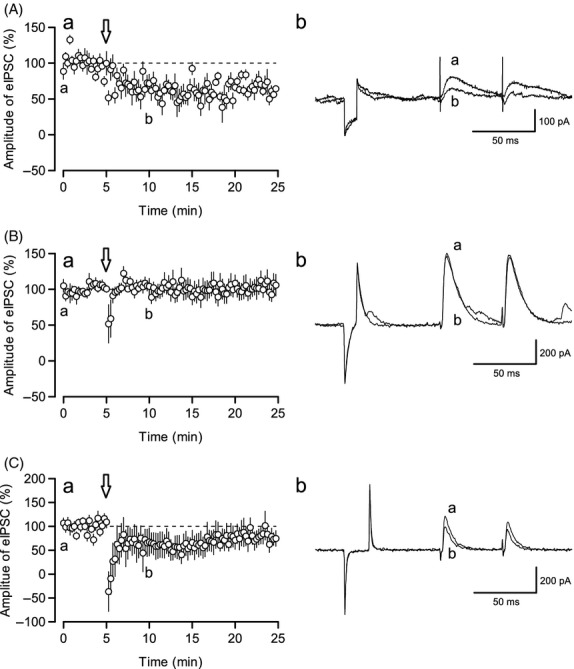
(A) a. Time course of the eIPSC amplitude recorded using gramicidin-perforated patch clamp recording (*n* = 5). Fifteen depolarizing train pulses (−64.3 mV to −14.3 mV for 1 sec at 0.5 Hz) were applied at *t* = 5, arrow; b. representative traces from this experiment recorded before (a, *t* = 0) and after (b, *t* = 10) PC depolarization in a (arrow). (B) a. Time course of the eIPSC amplitude recorded using a BAPTA-containing pipette solution (*n* = 9); b. representative traces of this experiment recorded before (a, *t* = 0) and after (b, *t* = 10) PC depolarization in a (arrow). (C) a. Time course of eIPSC amplitude in HEPES-ACSF (*n* = 9); b. representative traces of this experiment recorded before (a, *t* = 0) and after (b, *t* = 10) PC depolarization in a (arrow).

Originally, we observed DDI using a conventional whole-cell recording mode; thus, our observations may possibly be due to the artificial recording conditions. To address this concern, we attempted to record DDI using a gramicidin-perforated patch clamp mode (Eilers et al. 2001; Chavas and Marty 2003). Because the gramicidin-perforated patch clamp mode has a higher access resistance than conventional whole-cell voltage clamp mode, we applied a greater number of depolarizing pulses (15 pulses, −64.3 mV to −14.3 mV for 1 sec at 0.5 Hz). As shown in Figure 2A, eIPSC amplitudes during the long-lasting phase of DDI was significantly decreased following repetitive depolarizing pulses (before, 103.13 ± 10.47% at *t* = 3; after, 47.36 ± 10.97% at *t* = 20, *n* = 5, *P* < 0.05), but the PPR was not significantly affected (before, 1.48 ± 0.27 at *t* = 3; after, 1.03 ± 0.21 at *t* = 20, *n* = 5, *P* = 0.09). Finally, we examined whether DDI is observable at higher temperatures, which are closer to physiological conditions. When the recording temperature was increased to 30°C, we still consistently observed DDI (control, −32.30 ± 10.18% at *t* = 5, *n* = 8; 30°C, −6.93 ± 31.68% at *t* = 5, *n* = 3, *P* = 0.33, control, 62.51 ± 9.26% at *t* = 20, *n* = 8; 30°C, 54.55 ± 15.28% at *t* = 20, *n* = 3, *P* = 0.67, data not shown).

### [Ca^2+^]_i_ elevation induces DDI

**Figure 3:**
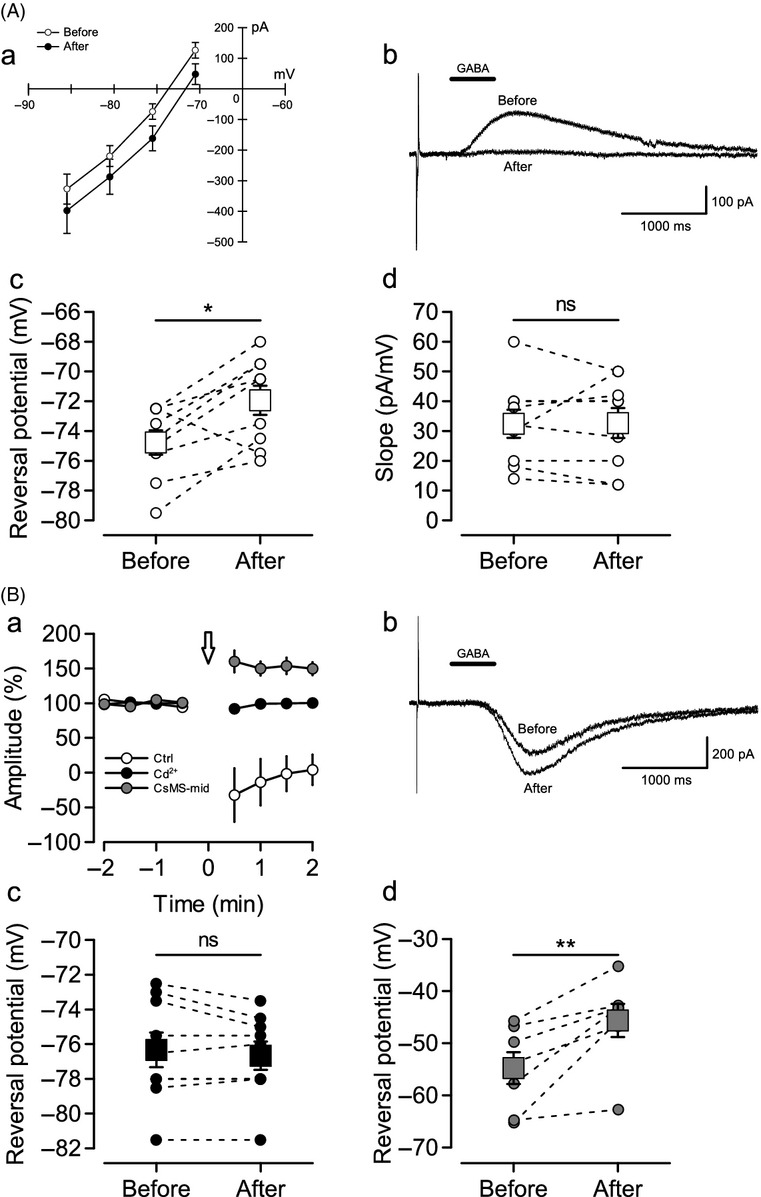
Depolarization of PCs induced [Cl^−^]_i_ elevation. (A) a. Current-voltage relationship of the GABA-mediated current following puff application of GABA (100 μmol/L for 500 msec) before (open circles) and after (filled circles) PC depolarization (*n* = 9). b. Representative traces of the GABA-induced current following puff application of GABA (100 μmol/L for 500 msec) before and after PC depolarization. c. Reversal potential of the GABA-induced current showed an obvious positive shift after PC depolarization (*P* < 0.05, *n* = 9). Each dashed line connecting the open circles represents data obtained from individual experiments. The squares indicate the mean ± SEM. d. Slope factors of the current-voltage relationship of the GABA-mediated current were not statistically different (*P* = 0.9, *n* = 9) before and after depolarization. Each dashed line connecting the open circles represents data obtained from an individual experiment. The squares indicate the mean ± SEM. (B) a. Time course of GABA_A_ receptor-mediated current recorded various conditions. TTX (0.5 μmol/L) and CGP 55845 (1 μmol/L) were added to ACSF. Five depolarizing train pulses (−70.5 mV to −20.5 mV for 1 sec at 0.5 Hz) were applied at *t* = 0, arrow. White circles indicate data obtained using the control pipette solution (*n* = 9). Black circles represent data obtained in the presence of Cd^2+^ to block VDCCs (100 μmol/L, *n* = 11). Gray circles indicate data recorded using the CsMS-mid pipette solution (*n* = 7). b. Representative traces of the GABA-induced currents recorded using CsMS-mid pipette solution. c. Reversal potential of the GABA-induced current in the presence of Cd^2+^ was not significantly different before and after PC depolarization (*P* = 0.24, *n* = 9). Each dashed line connecting the circles represents data obtained from an individual experiment. The squares indicate mean ± SEM. d. Reversal potential of the GABA-induced current recorded using CsMS-mid pipette solution showed an obvious positive shift following PC depolarization (*P* < 0.01, *n* = 7). Each dashed line connecting the circles represents data obtained from an individual experiment. The squares indicate mean ± SEM.

Membrane depolarization elicits an increase in the cytoplasmic Ca^2+^ concentration in PCs. Therefore, we next sought to determine whether DDI is triggered by intracellular Ca^2+^ elevation. We examined the effects of intracellular infusion of a fast Ca^2+^ chelator, BAPTA (5 mmol/L), on DDI. BAPTA injection into PCs via the recording pipette completely suppressed DDI (Fig. 2B). Compared with the data obtained using the normal internal solution (Fig. 1B), data obtained using BAPTA showed a significant difference both immediately after depolarization (control, −32.30 ± 10.18% at *t* = 5, *n* = 8; BAPTA, 51.74 ± 26.74% at *t* = 5, *n* = 9, *P* < 0.05) and during the long-lasting phase of DDI (control, 62.51 ± 9.26% at *t* = 20, *n* = 8; BAPTA, 108.49 ± 11.37%, at *t* = 20, *n* = 9, *P* < 0.01). Next, we tested whether VDCCs in the PC membrane are responsible for the [Ca^2+^]_i_ elevation. We recorded GABA-induced currents (stimulated by exogenous GABA, which was puff applied) in the presence and absence of 100 μmol/L cadmium (Cd^2+^) to block VDCCs (Fig. 3B a). To observe purely postsynaptic effects, we recorded the GABA_A_ receptor-mediated currents produced in response to a puff application of exogenous GABA (100 μmol/L for 500 msec) from a micropipette placed close to the primary dendritic shaft of the recorded PC. TTX (0.5 μmol/L) and CGP 55845 (1 μmol/L) were added to the ACSF to abolish spontaneous IPSCs and to block GABA_B_ receptor activation, respectively. When VDCCs were blocked, the depolarizing pulses failed to induce DDI (black circle, Fig. 3B a), indicating that DDI requires [Ca^2+^]_i_ elevation via VDCCs.

### Contribution of bicarbonate ions

It is well known that GABA_A_ receptors are permeable to bicarbonate ions as well as to Cl^−^ (Bormann et al. 1987); therefore, we also assessed the contribution of bicarbonate ion permeability to DDI. DDI was recorded in HEPES-ACSF to remove bicarbonate ions. As shown in Fig. 2C, DDI was evoked consistently, in spite of the lack of bicarbonate ions. There was no significant difference in the extent of depression of eIPSC amplitude between the control and HEPES-ACSF conditions (control, −32.30 ± 10.18% at *t* = 5, *n* = 8; HEPES-ACSF, −36.56 ± 41.26% at *t* = 5, *n* = 9, *P* = 0.92, control, 62.51 ± 9.26% at *t* = 20, *n* = 8; HEPES-ACSF, 71.09 ± 19.06% at *t* = 20, *n* = 9, *P* = 0.90, Fig. 1B), indicating it to be unlikely that bicarbonate ions are responsible for DDI.

### Depolarization of PCs causes [Cl^−^]_i_ elevation

To investigate the molecular mechanisms underlying DDI, we examined the reversal potential of GABA_A_ receptor-mediated currents and estimated the [Cl^−^]_i_ before and after PC depolarization. To avoid unexpected Ca^2+^ influx via VDCCs, the I-V relationship was obtained from the membrane potential at −70.5 mV to −85.5 mV, in 5-mV steps. The I-V relationship was recorded 2 min before and 2 min after PC depolarization (Fig. 3A a). It is noteworthy that, as shown in Figure 3A b, the amplitude of the GABA-mediated response was attenuated immediately after the depolarizing pulses in many cases (9 of 14 trials, 4.25 ± 21.88% of baseline). This suggests that the reduction in the amplitude of GABA-mediated responses was mediated postsynaptically. The reversal potential of GABA_A_ receptor-mediated currents showed a significant positive shift after the depolarizing pulses (before, −74.78 ± 0.81 mV; after, −71.94 ± 0.98 mV, *n* = 9, *P* < 0.05, Fig. 3A c). In contrast, we did not detect any significant change in the conductance of the GABA_A_ receptor Cl^−^ channels (before, 32.44 ± 4.73 pA/mV; after, 32.67 ± 5.04 pA/mV, *n* = 9, *P* = 0.93, Fig. 3A d). Further, there was no significant difference in either the reversal potential (before, −76.33 ± 1.00 mV; after −76.67 ± 0.82 mV, *n* = 9, *P* = 0.24, Fig. 3B c) or the conductance (before, 51.78 ± 7.64 pA/mV; after, 57.31 ± 9.27 pA/mV, *n* = 9, *P* = 0.20) of GABA_A_ receptor-mediated currents recorded in the presence of 100 μmol/L Cd^2+^. Furthermore, we examined the action of Cl^−^ on DDI (Fig. 3B). When we applied the DDI protocol with a higher [Cl^−^]_i_ (∼30 mmol/L, “CsMS-mid”, gray circle) pipette solution, as shown in Figure 3B a and B b, a marked potentiation of GABA_A_ receptor-mediated currents was observed, with a positive shift in the reversal potential of these currents (before, −54.07 ± 3.05 mV; after, −45.63 ± 3.17 mV, *n* = 7, *P* < 0.01, Fig. 3B d), but no accompanying shift in the conductance (before, 23.14 ± 3.75 pA/mV; after, 18.86 ± 2.46 pA/mV, *n* = 7, *P* = 0.29). Thus, it appears that the postsynaptic elevation of [Cl^−^]_i_ may be responsible for DDI.

### CaCC blockers abolish DDI

**Figure 4:**
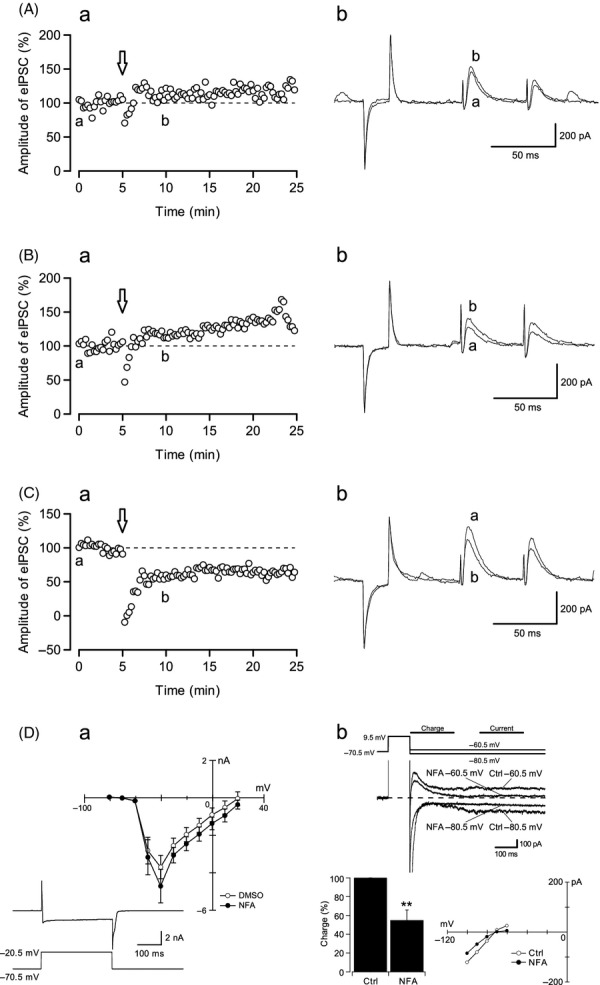
CaCC blockers disturbed DDI occurrence. (A) The experiments were performed using a pipette filled with NFA (10 μmol/L, *n* = 7), (B) DIDS (10 μmol/L, *n* = 8), and (C) vehicle (DMSO; 0.01%, *n* = 6). The left panel (a) represents the time course of the experiments. The right panel (b) shows representative traces from each experiment. The traces of a (before, at *t* = 0) and b (after, at *t* = 10) correspond to the timings shown on the left in the figures. Depolarizing trains were applied at *t* = 5, arrow. (D) a. Current-voltage relationships of calcium currents in the presence of NFA (filled circles, *n* = 8) and DMSO (open circles, *n* = 8). Data obtained from vehicle experiments with DMSO were used as a control. Inset; representative trace of voltage-dependent calcium current was recorded at a holding voltage of −70.5 mV to 20.5 mV with NFA in the pipette. b. Representative traces of chloride tail currents, elicited by holding voltages of −80.5 mV and −60.5 mV. NFA blocked the tail current of CaCCs (*n* = 8). Bar graph shows the effect of NFA on the charge of the tail current (*P* < 0.01, *n* = 8). The lower right graph shows the current-voltage relationships of tail currents in the absence and presence of NFA.

As neuronal [Cl^−^]_i_ is regulated by several ion channels and transporters (Stein and Nicoll 2003; Hartzell et al. 2005; Blaesse et al. 2009), we sought to determine which specific factors are involved in the alteration of [Cl^−^]_i_ in DDI. Because CaCCs contribute to the [Cl^−^]_i_ gradient in many different cell types, including airway epithelial cells, smooth muscle cells, and neuronal cells, as well as contributing to synaptic plasticity (Boucher et al. 1989; Llano et al. 1991; Hartzell et al. 2005), we investigated the effect of the CaCC blockers NFA and DIDS on DDI. When we recorded eIPSCs using an NFA (10 μmol/L)-containing pipette (Fig. 4A), the depolarizing pulses failed to induce both early (−9.30 ± 21.48% at *t* = 5, *n* = 6; NFA, 70.58 ± 11.98% at *t* = 5, *n* = 7, *P* < 0.01) and long-lasting phases of DDI (63.97 ± 18.55% at *t* = 20, *n* = 6; NFA, 127.3 ± 17.1% at *t* = 20, *n* = 7, *P* < 0.05). Similar results were obtained using DIDS (10 μmol/L)-containing pipette solution (Fig. 4B); there was a significant difference in both early (−9.30 ± 21.48% at *t* = 5, *n* = 6; DIDS, 47.06 ± 13.72% at *t* = 5, *n* = 8, *P* < 0.05) and long-lasting phases of DDI (63.97 ± 18.55% at *t* = 20, *n* = 8; DIDS, 142.37 ± 27.67% at *t* = 20, *n* = 8, *P* < 0.05). Conversely, DDI was observed when we used a control solution, containing the vehicle DMSO (Fig. 4C). DMSO did not affect DDI expression, with no significant difference in either the early (control, −32.30 ± 10.18% at *t* = 5, *n* = 8; DMSO, −9.30 ± 21.48% at *t* = 5, *n* = 8, *P* = 0.31) or the long-lasting phase of DDI (control, 62.51 ± 9.26% at *t* = 20, *n* = 8; DMSO, 63.97 ± 18.55% at *t* = 20, *n* = 6, *P* = 0.94). NFA was shown to have no effect on VDCCs that were activated by the same procedure used to induce DDI, that is, no difference was observed between currents induced by a command voltage of −20.5 mV in the presence and absence of NFA (DMSO, −1.96 ± 0.38 nA, *n* = 8; NFA, −2.45 ± 0.37 nA, *n* = 8, *P* = 0.37, Fig. 4D a). Furthermore, as shown in Figure 4D b, NFA significantly blocked CaCCs-activated tail currents at a command voltage of −50 mV (NFA, 54 ± 10.74% of control, *n* = 8, *P* < 0.01). Similar to NFA, even 10 μmol/L DIDS resulted in calcium currents induced by a brief voltage command (data not shown). Hence, our data indicate that CaCCs were primarily responsible for [Cl^−^]_i_ elevation following depolarization of PCs.

### Involvement of CCCs in [Cl^−^]_i_ alteration after PC depolarization

**Figure 5:**
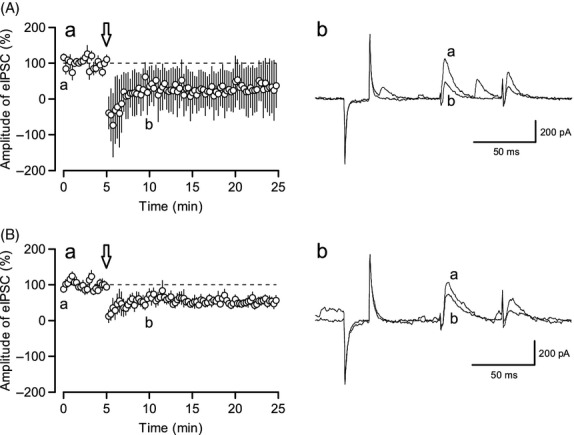
CCC inhibitors had little effect on DDI. The effects of DIOA (A. 5 μmol/L, *n* = 6) and bumetanide (B. 50 μmol/L, *n* = 7) on DDI. In the left panel, a shows the time course of eIPSC amplitude in the presence of DIOA and bumetanide. In the right panel, b shows representative traces from each experiment. Depolarizing pulses were applied at *t* = 5, arrow; a (before, at *t* = 0) and b (after, at *t* = 10) correspond to the timings shown in the left panels.

The increase in [Ca^2+^]_i_ following repetitive postsynaptic stimulation reportedly inhibits KCC2 function and decreases GABA-mediated inhibitory actions in hippocampal CA1 neurons (Fiumelli et al. 2005). Additionally, NKCCs play a role in the accumulation of cellular Cl^−^. Therefore, since KCC2s and NKCCs also potentially contribute to the [Cl^−^]_i_ change after PC depolarization, we examined whether DDI was affected by the CCC inhibitors DIOA (5 μmol/L) and bumetanide (50 μmol/L). Brain slices were incubated with ACSF containing 5 μmol/L DIOA, a KCC2 inhibitor, prior to the recordings. As shown in Figure 5A, DIOA treatment had no effect on DDI in either the early (control, −32.30 ± 10.18% at *t* = 5, *n* = 8; DIOA, −38.75 ± 44.68% at *t* = 5, *n* = 6, *P* = 0.87) or the long-lasting phase (control, 62.51 ± 9.26% at *t* = 20, *n* = 8; DIOA, 48.83 ± 71.15% at *t* = 20, *n* = 6, *P* = 0.83). However, the NKCC inhibitor bumetanide caused a weak blockage of DDI in the early phase (control, −32.30 ± 10.18% at *t* = 5, *n* = 8; bumetanide, 12.45 ± 17.72% at *t* = 5, *n* = 7, *P* < 0.05, Fig. 5B), but did not disturb the long-lasting phase (control, 62.51 ± 9.26%, at *t* = 20, *n* = 8; bumetanide, 59.45 ± 12.27% at *t* = 20, *n* = 7, *P* = 0.84, Fig. 5B). These results suggest that [Cl^−^]_i_ elevation in the early phase of DDI requires not only CaCC activation but also NKCC activation.

### CaMKII activation contributes to the DDI

**Figure 6:**
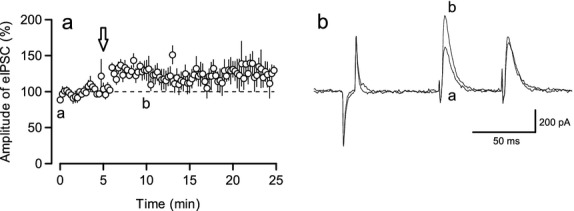
CaMKII inhibitor KN62 blocked DDI. (A) Time course of eIPSC amplitude in the presence of KN62 (3 μmol/L, *n* = 5). Depolarizing trains were applied at *t* = 5, arrow. (B) Representative traces from this experiment recorded before (a, *t* = 0) and after (b, *t* = 10) PC depolarization in a.

It has been reported that RP is blocked by a CaMKII inhibitor (Kano et al. 1996). To observe pure DDI, we obtained recordings in the presence of the CaMKII inhibitor KN62 (3 μmol/L, Fig. 6). We expected that KN62 would potentially enhance the extent of DDI by blocking the background activity of RP. However, contrary to our expectation, KN62 completely blocked DDI, both in the early (control, −32.30 ± 10.18% at *t* = 5, *n* = 8; KN62 103.39 ± 8.30% at *t* = 5, *n* = 5, *P* < 0.001) and the long-lasting phase (control, 62.51 ± 9.26%, at *t* = 20, *n* = 8; KN62 130.90 ± 10.93% at *t* = 20, *n* = 5, *P* < 0.001), indicating that CaMKII activation is required for not only RP but also DDI.

### CF stimulation reduces the extent of exogenous GABA-induced inhibition on spontaneous spike activity of PCs

**Figure 7:**
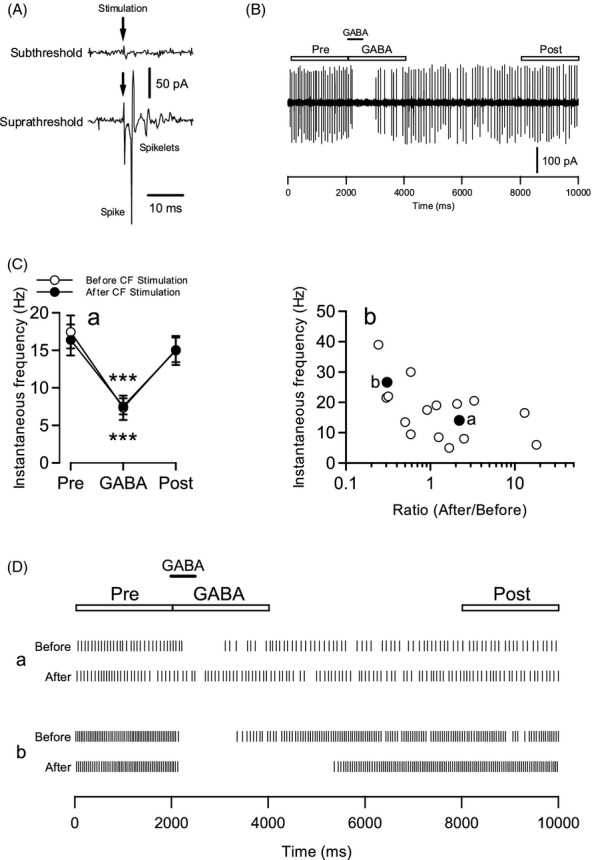
Inhibitory action of exogenous GABA (100 μmol/L for 500 msec) on spontaneous spike activity of PCs was modified by CF stimulation (1 Hz × 20 times). In this experiment, the direction of ACSF flow was set from the soma to the dendrites. (A) Representative responses to CF stimulation. Upper trace: subthreshold stimulation did not generate an action potential-derived spike. Lower trace: suprathreshold stimulation evoked a large spike, followed by small spikelets. The arrows indicate the timing of CF stimulation. (B) Representative data of GABA-induced inhibition of spike activity. The bar at the top represents the timing of GABA application. We defined 3 time windows, that is, pre (before GABA application), GABA (during GABA application), and post (after GABA application). Each window lasted 2000 msec. (C) a. GABA application decreased the instantaneous frequency of the spikes (*n* = 17). The instantaneous spike frequency was calculated for each time window, as described in B and D. Asterisks indicate statistically significant differences (***P < 0.001). The open and filled symbols indicate before and after CF stimulation, respectively. b. The scatter plot shows the relationship between the ratio of GABA-induced effective time and the instantaneous frequency of recorded PCs before CF stimulation (*n* = 17). Filled symbols correspond to the representative traces in D. (D) Raster plots of 2 types of modulatory actions, which represent time-shortened type in a and time-prolonged type in b. The raster plots of each cell show before (upper) and after (lower) CF stimulation. The bars and rectangles at the top represent the timing of GABA application and the time windows for the calculation of instantaneous spike frequencies, respectively**.**

Finally, we examined whether DDI also occurred under more physiological conditions, in the absence of any pharmacological agents. To this end, we recorded the spontaneous spikes of PCs in the cell-attached mode, using repetitive stimulation of CFs (1 Hz × 20 times) as the conditioning stimulus to depolarize PCs; the same paradigm as that demonstrated in Fig. 1D. The following CF-mediated responses were identified: (1) the stimulation-evoked spikes were subject to the all-or-none law in association with the stimulation intensity, and (2) the responses consisted of a large spike followed by a series of small spikelets (Fig. 7A). As shown in Figure 7B, spontaneous spike activity disappeared after the application of exogenous GABA (100 μmol/L for 500 msec) to the dendritic shafts of PCs (Fig. 7B). To quantitatively assess the GABA-induced inhibition of spike activity following CF stimulation, we defined 3 time windows and estimated the instantaneous frequency of the spikes in each period (Fig. 7B). We defined the extent of suppression of spikes during GABA application as the effect of GABA. The instantaneous frequency of spikes both before and after CF stimulation was not significantly different between the “pre” (time window of 2000 msec, immediately before application of exogenous GABA), and “post” (time window of 2000 msec, beginning 5500 msec after application of GABA) periods (before, pre, 17.44 ± 2.19 Hz; post, 15.06 ± 1.64 Hz, *n* = 17, *P* = 0.13; after, pre, 16.38 ± 2.08 Hz, post, 14.97 ± 1.94 Hz, *n* = 17, *P* = 0.24, Fig. 7C a) under each condition. In contrast, the instantaneous frequency of spikes during the GABA time window (2000 msec from beginning GABA application) was significantly attenuated (before, pre, 17.44 ± 2.19 Hz; GABA, 7.32 ± 1.62 Hz, *n* = 17, *P* < 0.001; after, pre, 16.38 ± 2.08 Hz; GABA, 7.53 ± 1.06 Hz, *n* = 17, *P* < 0.001, Fig. 7C a). Given that DDI is consistently induced by CF-driven depolarization of PCs, it was expected that the instantaneous spike frequency during GABA application would be increased. However, the average spike frequency of 17 neurons remained unchanged during the GABA time window when compared to the frequencies before and after CF stimulation (Fig. 7C a). In order to explore the impact of resting neuronal activity on CF-induced GABA-mediated actions, we plotted the relationship between the ratio of the spike frequency in the GABA time window (expressed as the ratio of the instantaneous frequency in the GABA window after CF stimulation to that before CF stimulation) and basal instantaneous frequency (in the “pre” time window) of recorded PCs before CF stimulation (Fig. 7C b). Linear regression analysis revealed a moderate negative correlation (*r* = −0.36, *n* = 17), but this was not statistically significant (*P* = 0.16).

Accordingly, as shown in the representative traces in Figure 7D, we found that GABA induced a lower (shortened type; *n* = 9, Fig. 7D a) or higher (prolonged type; *n* = 8, Fig. 7D b) instantaneous frequency in PCs after the conditioning stimuli, based on the basal instantaneous spike frequency of recorded PCs prior to CF stimulation. Therefore, not only suppression of GABA-induced inhibitory action (Fig. 7D a), but also enhancement of GABA-induced inhibitory action was caused by CF stimulation in a subset of PCs (Fig. 7D b). This enhancement of GABAergic inhibitory action is likely to be due to RP (Kano et al. 1992). Finally, in order to estimate the effects of DDI on tonic inhibition, we calculated the coefficient of variation (CV) in the pre time window of the interspike intervals, between before and after DDI induction (Häusser and Clark 1997). The CV of the interspike intervals was 0.55 ± 0.17 (before) and 0.73 ± 0.21 (after) in the pretime window. Although, on average, the CV was reduced by DDI, there was no significant difference (shortened type, *P* = 0.50, *n* = 9). We used rats younger than those used in a previous study (P18–32, Häusser and Clark 1997), and the extent of the inhibitory input might be smaller in younger animals. Actually, the frequency of sIPSCs increased developmentally during the second postnatal week and reached a plateau at around P15 in rat cerebellar PCs (Casel et al. 2005). Taken together, these results support the hypothesis that, at least in a part of the cerebellar cortex, CF-induced DDI of PCs played a role in the disinhibition of GABA-mediated inhibition.

## Discussion

This study demonstrates the occurrence of DDI in cerebellar PCs under conditions that elicited outward GABAergic eIPSCs (Fig. 1A). According to our results, the PPR was not changed before and after PC depolarization (Fig. 1C), and the reversal potential of the GABA_A_ receptor-mediated current was shifted positively (Fig. 3A). In addition, we found that both BAPTA (Fig. 2B) and Cd^2+^ blocked DDI (Fig. 3B). Therefore, DDI appears to be induced by a postsynaptic mechanism that causes a decrease in the driving force of Cl^−^ through the Ca^2+^-dependent elevation of postsynaptic [Cl^−^]_i_ in PCs.

In this study, we used relatively young rats (postnatal days 11–15). Many reports have demonstrated that the neuronal concentration of intracellular Cl^−^ changes during development (Rivera et al. 1999; Ben-Ari 2002). With regards to rat cerebellar PCs, the reversal potential of GABA-induced current shifted during development to more negative values, and finally equilibrated at −87 mV after P15 (Eilers et al. 2001). Eilers et al. (2001) reported the reversal potential of GABA-induced current to be approximately −85 mV after P12. Therefore, it is likely that the [Cl^−^]_i_ in the PCs used in this study had already attained the almost steady-state [Cl^−^]_i_ level of mature PCs. The estimated equilibrium potential for Cl^−^ is −87 mV under our experimental conditions. In contrast, we observed that the reversal potential of GABA_A_ receptor-mediated current was −74.78 mV (Fig. 3A). This discrepancy between the theoretical and measured values was likely due to the contribution of a persistent, unknown Cl^−^ conductance. The resting membrane potential of PCs in this study was −62.79 ± 0.99 mV (*n* = 9). Therefore, these results strongly suggest that GABA-mediated current acts as an inhibitory current, since the reversal potential of this current was found to be more hyperpolarized than the resting membrane potential.

**Figure 8:**
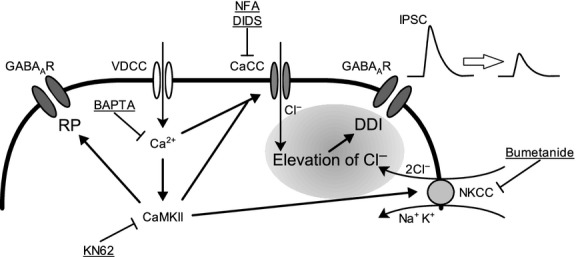
Schematic diagram of postsynaptic depolarization-induced plasticity of inhibitory transmission in PCs. PC depolarization elicits Ca^2+^-influx via VDCCs. The elevation of [Ca^2+^]_i_ activates both CaCCs and CaMKII. The activated CaMKII induces RP and regulates the activities of CaCCs and NKCCs. Activated CaCCs and NKCCs lead to alteration in [Cl^−^]_i_. The resulting [Cl^−^]_i_ elevation induces DDI. Underlines represent chemicals used in this study to block or chelate specific molecules in this pathway.

In pharmacological experiments, the CaCC blockers NFA and DIDS abolished DDI (Fig. 4). Furthermore, DDI was partially blocked by the NKCC inhibitor bumetanide, but not by the KCC2 inhibitor DIOA (Fig. 5). These results suggest that the activation of both CaCCs and NKCCs is necessary for [Cl^−^]_i_ change after PC depolarization. Our findings indicate that CaCCs were the key molecules involved. It has been previously reported that Ca^2+^-dependent Cl^−^ conductance in PCs was recorded as tail currents following the activation of VDCCs (Llano et al. 1991). The expression of at least 2 types of CaCC isoforms has been described in the rat cerebellum using an RT-PCR technique (Yoon et al. 2006). Therefore, it is conceivable that DDI, which is caused by the change in the [Cl^−^]_i_ following CaCC activation, occurs under physiological conditions. Considering that DDI was observed in the whole-cell patch clamp mode, CaCCs might be possibly localized very close to GABA_A_ receptors on the plasma membrane and sense the localized [Cl^−^]_i_ change in a limited cytoplasmic area. Furthermore, DDI was blocked by the CaMKII inhibitor KN62 (Fig. 6). This finding indicates that CaMKII is also a key molecules in modulating the PC GABAergic transmission postsynaptically (Fig. 8). Finally, we demonstrated that, in approximately 50% of the PCs, stimulation of CFs onto PCs reduced the effect of GABA-induced inhibition on spontaneous spike generation in the PCs (Fig. 7).

Collectively, our findings suggest that DDI is a novel, GABAergic form of synaptic plasticity in cerebellar PCs and is distinct from other postsynaptic mechanisms, which have been well established as depolarization-induced plasticity, for example, RP (Kano et al. 1992).

### DDI is generated postsynaptically

It has been previously reported that repetitive depolarizing pulses enhance the amplitude of stimulation-evoked IPSCs (Duguid and Smart 2004), a phenomenon which was termed DPI. It has also been demonstrated that RP-like IPSC potentiation can be recorded using a low Cl^−^-containing pipette solution (Duguid et al. 2007). Since our recording conditions included APV to block DPI, we were able to discriminate between the components of DPI and DDI. It is well known that repetitive CF stimulation suppresses GABAergic inhibitory transmission between interneurons and PCs through the activation of presynaptic AMPA receptors (Satake et al. 2000, 2004). Furthermore, it has been reported that repetitive depolarizing pulses induce retrograde dopamine release from PCs, evoking the depolarization-induced slow current DISC (Kim et al. 2009). Our findings clearly discriminate DDI from both the AMPA receptor-mediated inhibition of GABAergic transmission and the possibility of a contribution of retrograde dopamine release for the following reasons. First, under our experimental conditions, the ACSF contained the AMPA/kainate receptor antagonist CNQX. Second, the PPR was not significantly different before and after PC depolarization. Third, several of the protocols used in our study employed puff application of exogenous GABA, which rules out the contribution of presynaptic transmission (Figs. 3A and 7). Fourth, presynaptic inhibition of GABAergic transmission, such as with DSI, did not elicit a change in the direction of eIPSCs (Fig. 1A). Finally, we sought to examine whether the retrograde release of dopamine affects DDI. Our preliminary study using bath application of dopamine showed a slight enhancement of eIPSC amplitude (*n* = 4, unpublished data). This dopaminergic effect was in the opposite direction to the effect of DDI, indicating that DDI is not affected by the retrograde release of dopamine.

### Implications of depolarization for synaptic depression and potentiation

We demonstrated here that the depolarization of PCs attenuated the amplitude of eIPSCs under both conventional and perforated patch-clamp conditions (Figs. 1 and 2A). A previous study, which also used slice preparations, reported that depolarization-induced Ca^2+^ entry enhances the amplitude of exogenous GABA-induced currents and reduces inhibitory synaptic currents in PCs (Llano et al. 1991). Other groups have also reported that mGluR1 activation enhances exogenous GABA-induced currents in cultured PCs (Hashimoto et al. 1996; Sugiyama et al. 2008). All these previous studies used a high-Cl^−^ pipette solution when recording GABA-induced currents. Here, we demonstrated that postsynaptic depolarizing pulses potentiated the amplitude of GABA_A_ receptor-mediated currents using a CsMS-mid pipette solution, containing a moderate concentration of Cl^−^ (Fig. 3B a, gray circle). Therefore, it is likely that PC depolarization increases [Cl^−^]_i,_ and thus affects the amplitude of GABAergic transmission in PCs. In our experiments, the potentiation of eIPSC amplitude was also observed following the depolarizing pulses (Fig. 4A and B) in the presence of CaCC blockers. Because these blockers inhibited [Cl^−^]_i_ elevation and prevented any change to the driving force for Cl^−^ influx after PC depolarization, we were able to observe RP-like potentiation of GABAergic transmission.

It is well established that RP is suppressed by the CaMKII inhibitor KN62 (Kano et al. 1996). In order to isolate DDI from RP, we performed an experiment in the presence of KN62. When CaMKII was inhibited, depolarized PCs failed to show DDI, and there was, in fact, a slight potentiation of the eIPSC amplitude (Fig. 6). These results suggest that CaMKII is tightly coupled to CaCC activity. In support of this notion, it has been reported that CaCCs are directly activated by CaMKII in a human pancreatoma epithelial cell line (Ho et al. 2001), intestinal smooth muscle (He and Goyal 2012), and recombinant CLC-3 chloride channels (Robinson et al. 2004). Furthermore, in the DDI signaling pathway, intracellular Ca^2+^ elevation via VDCCs is necessary for CaMKII activation; this was confirmed when the use of Cd^2+^-containing ACSF, and the intracellular application of BAPTA inhibited DDI. However, PC depolarization elicited a low-level potentiation of eIPSCs when CaMKII was inhibited (Fig. 6). This potentiation was likely due to CaMKII-independent RP. Actually, as shown in Figure 7, GABA-induced inhibition of spontaneous spike generation varied after CF stimulation. Contrary to our expectations, ∼50% of the recorded neurons showed enhancement of GABA-mediated inhibition (Fig. 7D). Thus, it is likely that both RP and DDI are induced in a similar time frame following PC depolarization and are dependent on [Cl^−^]_i_ alteration (Fig. 8).

### Physiological roles of DDI

It remains unclear what factors induce DDI under physiological conditions. PCs exhibit heterogeneous effects of GABA-mediated inhibition following CF stimulation, namely DDI and RP. DDI after the depolarizing pulses was observed in almost all cases under voltage clamp conditions. Therefore, all PCs may possess the potential to show DDI after depolarization. Regression analysis showed a moderate correlation between the ratio of the GABA-induced effective time and the instantaneous frequency of recorded PC spike before CF stimulation (Fig. 7C b). In fact, it seems that PCs with a lower instantaneous spike frequency are more likely to show DDI, while PCs with a higher spontaneous frequency are more likely to show RP (Fig. 7D). Since spontaneous spike activity is closely related to [Ca^2+^]_i_ (Sugimori and Llinás 1990), resting [Cl^−^]_i_ immediately before CF excitation might be a determining factor for the expression of the 2 types of postsynaptic plasticity (Fig. 8). That is, spontaneous spikes induce [Ca^2+^]_i_ elevation, which promotes the alteration of [Cl^−^]_i_ in PCs. Taken together, DDI and RP may play important roles in reciprocally regulating activity within the neuronal networks of the cerebellar cortex in a PC state-dependent manner.
